# The utilization of transperineal ultrasound following fetal heart deceleration after epidural analgesia: a case report

**DOI:** 10.1186/s12884-022-05197-7

**Published:** 2022-11-24

**Authors:** Shimiao Feng, Juan Gu

**Affiliations:** 1 Department of Anesthesiology, West China Second University Hospital, Sichuan University, 20#, Section 3 Renmin Nan Road, Chengdu, Sichuan 610041 China; 2 Key Laboratory of Birth Defects and Related Diseases of Women and Children (Sichuan University), Ministry of Education, 20#, Section 3 Renmin Nan Road, Chengdu, Sichuan 610041 China

**Keywords:** Epidural analgesia, Transperineal ultrasound, Fetal heart rate, Case report

## Abstract

**Background:**

We report a case of fetal heart rate decelerations and relaxation of pelvic muscles and fetal descent using transperineal ultrasound after initiation of epidural labor analgesia.

**Case presentation:**

A 32-year-old woman, G1P0 with gestational age of 40 weeks, required epidural analgesia when her cervical dilatation was 2 cm. Baseline transperineal ultrasound examination was performed before epidural puncture. The fetal heart rate tracing was normal before the initiation of analgesia. Approximately 10 min after the epidural administration of the loading dose, the patient reported onset of analgesia and the FHR tracing showed variable-decelerations. There was no hypotension or evidence of uterine tachysystole. Transperineal ultrasound was performed again after epidural analgesia took effect. The anteroposterior diameter of the levator hiatus increased from 5.3 to 6.6 cm and angle of progress increased from 116°to 133°. The relaxation of pelvic muscle and rapid descent of fetal head may have contributed to the FHR deceleration. The midwife elevated the fetal head through the vagina with her hand, and the FHR recovered soon thereafter.

**Conclusions:**

Changes in fetal heart rate after initiation of neuraxial analgesia are unpredictable. In addition to FHR and tocodynametric monitoring, performing TPU may helpful in distinguishing the reasons for FHR change and initiating corresponding corrective measures.

## Background

A lot studies have reported anomalies in the fetal heart rate (FHR) following epidural analgesia [1, 2]. The most common abnormity is FHR deceleration. Although there is no impact on neonatal clinical state, it may increase the probability of cesarean delivery and decrease the umbilical artery PH. The mechanisms of epidural analgesia induce FHR deceleration have also been explored, which mainly focus on the maternal hypotension and uterine hypertonus [3]. Interventions like change of maternal position, administration of supplemental oxygen, correction of hypotension and administration of tocolytic agents are suggested to facilitate recovery of the FHR. Many other factors may be related to the FHR deceleration after epidural analgesia, such as rapid fetal head decent caused by uterine stimulation or relaxation of perineal muscles. We report a case of FHR decelerations and relaxation of pelvic muscles and fetal descent using transperineal ultrasound (TPU) after initiation of epidural labor analgesia.

## Case presentation

A 32-year-old woman, 168 cm, 68 kg, G1P0 with gestational age of 40 weeks, required epidural analgesia when her cervical dilatation was 2 cm. She has gestational diabetes mellitus which was controlled well. Her prenatal visit was normal and her other medical history was un-markable. She had regular uterine contraction without rupture of membranes and the contractions interval was about 3 min. The fetal position was ROA (Right Occipito-Anterior) and the biparietal diameter was 9.4 cm.

**Fig. 1:**
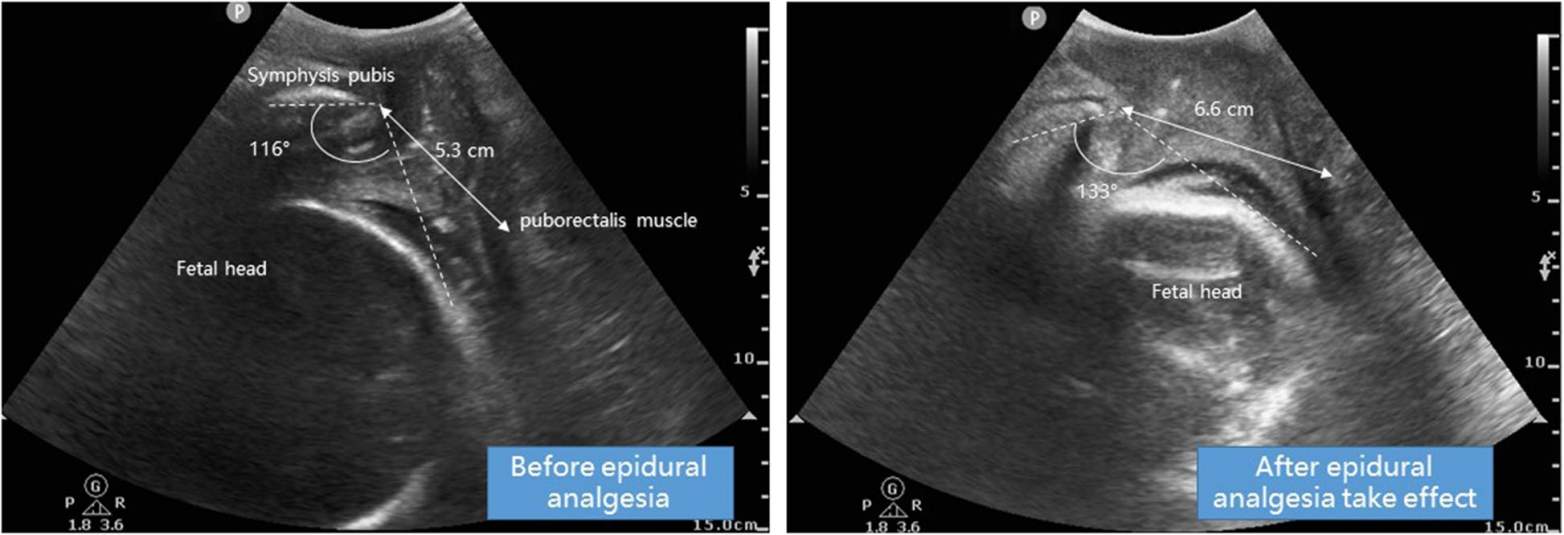
The variation of transperineal ultrasound measured at rest with fetal heart deceleration associated with initiation of epidural labor analgesia. Transperineal ultrasound (TPU) was performed before epidural analgesia and after epidural analgesia was attained. The anteroposterior diameter of the levator hiatus (APD) increased from 5.3 to 6.6 cm and angle of progress (AoP) increased from 113° to 133°

After she arrived at the operational room for epidural analgesia, routine monitoring was performed and 500 ml lactated Ringer's solution was administrated. Blood pressure was 120/75 mmHg, heat rate was 85 beats per minute, pulse oxygen saturation was 98%. Baseline TPU examination was performed just before epidural puncture in a study of our team as previously described [4]. A convex transducer 3.0–5.0 MHz (Philips CX50) was placed in the perineum in the midsagittal plane, the midsagittal view was obtained at rest using the following landmarks (Fig. 1): pubic symphysis, fetal head, vagina, rectum and puborectalis muscle. The fetal heart rate tracing was normal before the initiation of analgesia. Epidural puncture was finished within 5 min and a loading dose of 0.75% lidocaine 15 ml was administrated. Approximately 10 min later, the patient reported onset of analgesia and the FHR tracing showed variable-decelerations. There was no hypotension or evidence of uterine tachysystole. Blood pressure was 106/70 mmHg, the contractions interval was 2 min. TPU was performed again after epidural analgesia took effect. The anteroposterior diameter of the levator hiatus (APD) increased from 5.3 to 6.6 cm and angle of progress (AoP) increased from 116°to 133°. Using the equation, fetal presentation (cm) = AoP (◦) × 0.0937 -10.911 [5], the fetal head was calculated to have descended approximately 1.6 cm. The relaxation of pelvic muscle and rapid descent of fetal head may have contributed to the FHR deceleration. The midwife elevated the fetal head through the vagina with her hand, and the FHR recovered soon thereafter. The baby delivered uneventfully with 12 h of epidural analgesia.

## Discussion and conclusions

TPU is objective, accurate and reproducible for the evaluation of the pelvic floor function and labor progress [5]. APD has a good correlation with the tension of the levator ani muscle, and AoP can objectively and precisely reflect the fetal head station in the birth canal [6, 7]. Rapid fetal head descent from relaxation of pelvic muscles may be another reason, except for uterine hypertonus and maternal hypotension, for FHR changes following initiation of neuraxial analgesia. We used TPU to document relaxation of the pelvic musculature and rapid fetal head descent in this case. The fetal head descended about 1.6 cm within 15 min, it is significantly exceeds the rate of median time for descent at first stage reported by previous study [8]. Rapid descent caused FHR deceleration may due to head compression by un-dilated cervix even between contractions [9, 10]. Fetal heart rate after initiation of neuraxial analgesia are unpredictable, so, it is important to timely find out the accurate reason. Is hypotension, hypertonus or rapid decent? Hypotension and hypertonus can be easily observed by the commonly used blood pressure monitoring and electronic fetal heart monitoring after epidural analgesia. On these basis, TPU is a useful tool used to find the rapid fetal head decent after onset of epidural analgesia. And the accordingly interventions should be taken to facilitate recovery of the FHR.

We suggest that elevating the fetal head through the vagina may be an effective method to facilitate recovery of the FHR. The purpose of pushing of the fetal head with a vaginal hand we suggest in our case is to prevent the rapid decent of fetal head, relive the head compression and facilitate recovery of the FHR. However, the risks associated with this procedure, such as neonatal skull fracture, should also be considered [11, 12]. But, the power needed to push the fetal head we suggest is less than those mentioned in the context of an impacted fetal head and obstructed labor, and the risks of fetal injury may be less.

In conclusion, changes in fetal heart rate after initiation of neuraxial analgesia are unpredictable. In addition to FHR and tocodynametric monitoring, performing TPU may helpful in distinguishing the reasons for FHR change and initiating corresponding corrective measures.

## Data Availability

The datasets used and/or analysed during the current study are available from the corresponding author on reasonable request.

## Abbreviations

FHR: Fetal heart rate
TPU: Transperineal ultrasound
ROA: Right Occipito-Anterior
APD: Anteroposterior diameter of the levator hiatus
AoP: Angle of progression
